# Analysis of risk factors for prolonged intensive care unit stay after colorectal cancer surgery

**DOI:** 10.3389/fonc.2025.1620741

**Published:** 2026-01-12

**Authors:** Xuefeng Song, Yabin Liu, Biao Dong

**Affiliations:** 1 Department of Gastrointestinal Oncology, Shanxi Province Cancer Hospital/Shanxi Hospital Affiliated to Cancer Hospital, Chinese Academy of Medical Sciences/Cancer Hospital Affiliated to Shanxi Medical University, Taiyuan, Shanxi, China; 2 Department of the Hepatobiliary Surgery Fourth Hospital of Hebei Medical University, Shijiazhuang, Hebei, China

**Keywords:** colorectal cancer, ICU length of stay, predictive, risk factors, surgery

## Abstract

**Objective:**

This study employed a retrospective analysis to investigate the risk factors associated with prolonged intensive care unit (ICU) stay following colorectal cancer surgery.

**Methods:**

A total of 325 patients who underwent colorectal cancer surgery and were subsequently transferred to the ICU at the Fourth Hospital of Hebei Medical University were enrolled as the colorectal cancer group. The ICU length of stay (LOS) was stratified by quartiles, with the 75th percentile (≥61 hours) defined as the prolonged ICU stay group. Basic information, preoperative comorbidities, surgical details, and postoperative complications were compared between the two groups. Univariate and multivariate analyses were performed to identify risk factors for prolonged ICU stay. The predictive ability of the model was evaluated using the area under the receiver operating characteristic (ROC) curve (AUC). Calibration curves were used to assess the agreement between predicted and observed outcomes, and decision curve analysis (DCA) was conducted to evaluate the net benefit for patients.

**Results:**

Among the 325 enrolled patients (median age 77 years), 183 (56.3%) were male and 142 (43.7%) were female. The cohort was divided into a normal group (n = 243) and a prolonged group (n = 82). Univariate analysis identified preoperative obstruction, preoperative perforation, Class IV surgical incisions, open surgical approach, intraoperative blood loss, duration of mechanical ventilation, perioperative sepsis, perioperative acute kidney injury (AKI), lower extremity deep vein thrombosis (DVT), and mean postoperative platelet count, albumin, and blood urea nitrogen levels within the first 24 hours of ICU admission as risk factors for prolonged ICU stay. After adjusting for confounding factors, multivariate logistic regression analysis revealed that the laparotomy, perioperative sepsis, postoperative duration of mechanical ventilation, occurrence of lower extremity DVT, and mean platelet count within the first day of ICU admission were independent risk factors for prolonged ICU stay. The AUC of the ROC curve was 0.8081 (95% confidence interval [CI]: 0.745–0.870), indicating strong discriminatory ability. The calibration curve demonstrated excellent agreement between predicted and observed outcomes (observed-to-expected [O:E] ratio = 1.000, calibration-in-the-large [CITL] = -0.000, slope = 1.000). Bootstrap validation yielded a Brier score of 24.1%, a concordance statistic (C-statistic) of 0.772, an E:O ratio of 0.981, a slope of 0.756, and a CITL of 0.029. DCA revealed a high net benefit for predicting prolonged ICU stay at lower threshold probabilities. Subgroup analysis by surgical site (left-sided vs. right-sided colon cancer) showed AUCs of 0.7892 (95% CI: 0.71–0.87) for left-sided colon cancer and for right-sided colon cancer of 0.8253 (95% CI: 0.72–0.93).

**Conclusion:**

The laparotomy, perioperative sepsis, postoperative duration of mechanical ventilation, occurrence of DVT, and mean platelet count within the first day of ICU admission were independent risk factors for prolonged ICU stay following colorectal cancer surgery.

## 1 Introduction

Colorectal cancer (CRC) was one of the most prevalent malignant tumors globally, with consistently high morbidity and mortality rates (1). Surgical resection remains the primary treatment modality for CRC (2), yet the postoperative recovery trajectory varies significantly among patients due to individual differences. In recent years, the widespread adoption of the Enhanced Recovery After Surgery (ERAS) paradigm has markedly shortened postoperative hospital stays, reduced healthcare costs, potentially improved surgical outcomes, and mitigated complications. This approach has not only shortened the length of stay (LOS) but also enhanced patient satisfaction (3). However, in clinical practice, a subset of patients experiences prolonged postoperative hospitalizations, which not only escalates the consumption of medical resources but may also adversely impact patient prognosis. Moreover, extended hospitalizations exert negative influences on medical costs, healthcare workflows, and, importantly, on the patients themselves, owing to heightened risks of nosocomial infections and deterioration of the hospital environment (4).

Postoperative ICU management is indicated for patients with severe complications or unstable vital signs. The main goals of ICU care include close monitoring of vital parameters, organ function maintenance, and provision of supportive therapies such as respiratory support, circulatory stabilization, and infection control. Colorectal cancer (CRC) patients with comorbidities are at increased risk of postoperative complications, which can result in prolonged hospital stays, higher healthcare costs, delays in adjuvant therapies, and elevated mortality (5). In Canada, 17.7% of postoperative CRC patients are transferred to the ICU, the highest rate among non - cardiac surgeries (6). Evidence shows that postoperative ICU admission reduces mortality in CRC patients (7). The reasons for ICU admission in these patients include surgical risks, patient - specific risks such as cardiovascular, renal, neurological (e.g., delirium) conditions, and the unpredictability of major postoperative complications (8).

The duration of ICU stay following CRC surgery serves as a crucial indicator of both the quality of postoperative recovery and the efficiency of medical resource utilization. Prolonged ICU stays not only reflect the risk and severity of postoperative complications but are also intimately linked to the consumption of medical resources, patients’ quality of life, and escalating healthcare costs. Shortening the ICU LOS is a cornerstone objective of the ERAS philosophy, contributing to the reduction of postoperative complications, lowering infection risks, enhancing the efficiency of medical resource utilization, and alleviating the economic burden on patients and their families. Furthermore, attention to prolonged ICU stays facilitates the identification of high-risk patients, the optimization of postoperative management strategies, and the delivery of more precise, individualized treatment plans. Therefore, a thorough analysis of the risk factors contributing to prolonged ICU stays following CRC surgery holds significant implications for improving patient outcomes, enhancing medical quality, and advancing the implementation of the ERAS concept. While current research predominantly focuses on postoperative hospital stays, studies specifically addressing ICU LOS are scarce. Consequently, the objective of this study is to analyze, within a retrospective cohort of CRC patients admitted to the ICU postoperatively, the risk factors associated with prolonged ICU stays. This endeavor is of paramount importance for optimizing postoperative management and elevating medical quality.

## 2 Methods

### 2.1 Study population

This is a single-center, retrospective study. A total of 325 patients who underwent colorectal cancer surgery and were subsequently transferred to the ICU with tracheal intubation at our hospital between May 2020 and January 2022 were included.

### 2.2 Inclusion and exclusion criteria

#### 2.2.1 Inclusion criteria

Pathologically confirmed diagnosis of colorectal cancer who were transfer to the ICU after surgery. The interquartile range (IQR) for ICU length of stay (LOS) was determined to be 25 (21, 61) hours. Patients with an ICU LOS ≥ 61 hours were categorized into the prolonged group. Consequently, the cohort was divided into a normal group (n=243) and a prolonged group (n=82).

#### 2.2.2 Exclusion criteria

Patients transferred back to the general ward immediately after surgery without ICU admission.

### 2.3 Ethics statement

This study was approved by the Ethics Committee of the Fourth Hospital of Hebei Medical University (Ethics Approval Number: 2022KY094). All enrolled patients provided informed consent, either personally or through their legal representatives.

### 2.4 Data collection

Baseline demographic characteristics (gender, age, height, weight) and comorbidities were recorded. ICU admission and discharge times were documented. The following clinical information was also collected.

TNM staging of the tumor. Presence of preoperative bowel obstruction or perforation.

Surgical approach (laparoscopic vs. open surgery), incision type, and intraoperative blood loss. Perioperative complications, including sepsis, wound infection, intestinal fistula, wound dehiscence, acute kidney injury, and pulmonary infection.

The average values of laboratory test results obtained within the first 24 hours of ICU admission were recorded.

### 2.5 Statistical analysis

Data management and statistical analysis were performed using Stata 18 software. Normally distributed data were expressed as mean ± standard deviation $\left(\bar{\text{x}}\pm \text{s}\right)$, and comparisons between groups were conducted using the independent samples t-test. Non-normally distributed data were presented as median (M) with interquartile range (Q1, Q3), and comparisons between groups were performed using the Wilcoxon rank-sum test. Categorical data were expressed as frequencies (percentages), and intergroup comparisons were analyzed using the chi-square test or Fisher’s exact probability method, as appropriate. To identify risk factors associated with prolonged transfer from the ICU in postoperative colorectal cancer patients, covariates with a P-value< 0.05 in univariate analysis were included in the multivariate logistic regression model. Collinearity among covariates was assessed using variance inflation factors (VIFs) and tolerance coefficients. The area under the receiver operating characteristic (ROC) curve (AUC) was used to evaluate the predictive ability for prolonged ICU stay. Calibration curves were constructed to assess the agreement between predicted and observed outcomes. Decision curve analysis (DCA) was employed to evaluate the net benefit to patients. All statistical tests were two-tailed, and a P-value< 0.05 was considered statistically significant.

## 3 Results

### 3.1 Baseline characteristics

Among the 325 enrolled patients (median age 77 years), 183 (56.3%) were male and 142 (43.7%) were female. There were no statistically significant differences between the normal group and the prolonged group in terms of diabetes, coronary heart disease, arrhythmia, cerebrovascular disease, or chronic kidney disease. Similarly, no significant differences were observed in tumor staging (TNM stages 1, 2, 3, and 4) between the two groups (**Table 1**).

**Table 1:** Comparison of patients’ basic information.

Variable	ALL (N = 325)	Normal group (N = 243)	Delayed group (N = 82)	X^2^/t	p
Age	77(68,83)	76(68,82)	78(66,83)	0.091	0.76
Gender(M, %)	183(56.3%)	139(57.2%)	44(53.66%)	0.31	0.58
BMI	23.24(20.76,25.43)	23.3(21.23,25.39)	2.96(20,25.86)	0.74	0.39
Hypertension(n,%)	138(42.46%)	105(43.21%)	33(40.24%)	0.22	0.638
Diabetes(n,%)	81(24.92%)	56(23.05%)	15(18.29%)	0.81	0.37
coronary heart disease(n,%)	87(26.77%)	70(28.81%)	17(20.73%)	2.04	0.15
Arrhythmia(n,%)	18(5.54%)	11(4.53%)	7(8.54%)	1.88	0.17
Cerebrovascular disease(n,%)	27(8.31%)	18(4.53%)	9(10.98%)	1.02	0.311
CKD(n,%)	7(2.15%)	7(2.88%)	0(0%)	2.41	0.12
TNM-1(n,%)	14(4.31%)	13(5.35%)	1(1.2%)	2.54	0.11
TNM-2(n,%)	116(35.69%)	85(34.98%)	31(37.8%)	0.21	0.64
TNM-3(n,%)	112(34.46%)	86(35.39%)	26(31.71%)	0.37	0.54
TNM-4(n,%)	74(22.77%)	55(23.05%)	19(23.17%)	0.01	0.92

### 3.2 Surgical outcomes

Surgical results are detailed in **Table 2**.

**Table 2:** Comparison of patient surgical information.

Variable	ALL (N = 325)	Normal group (N = 243)	Delayed Group (N = 82)	Z/t	p
Preoperative obstruction(n, %)	35(10.77%)	21(8.64%)	14(17.07%)	4.54	0.033
Preoperative perforation(n, %)	21(6.46%)	10(4.12%)	11(13.41%)	8.77	0.003
Surgical site - left half(n, %)	108(33.23%)	81(33.33%)	27(32.93%)	0.005	0.946
Surgical site - right half(n, %)	217(66.77%)	162(66.67%)	55(67.07%)	0.005	0.946
Surgical incision type III(n, %)	19(5.85%)	12(4.94%)	7(8.54%)	1.44	0.23
Surgical incision type IV(n, %)	14(4.31%)	6(2.47%)	8(9.76%)	7.9	0.005
Surgical approach - laparotomy(n, %)	97(29.85%)	56(23.05%)	41(50%)	21.27	0.001
Intraoperative bleeding(n, %)	50(50,100)	50(40,50)	50(50,100)	4.34	0.03
Ventilation machine time(h)	16(13,20)	15(13,18)	20.5(15,71)	24.86	0.001
Perioperative sepsis(n, %)	25(7.69%)	4(1.65%)	21(25.61%)	27.99	0.001
Perioperative AKI(n, %)	15(4.62%)	3(1.23%)	12(14.63%)	19.18	0.001
Postoperative fistula(n, %)	6(1.85%)	4(1.65%)	2(2.44%)	0.213	0.645
DVT(n, %)	40(12.31%)	24(9.88%)	16(19.51%)	5.27	0.02
Postop wound infection(n, %)	18(5.54%)	13(5.35%)	5(6.10%)	0.065	0.798
Postoperative wound dehiscence(n, %)	8(2.46%)	5(2.06%)	3(3.66%)	0.65	0.42
Postoperative pulmonary infection(n, %)	10(3.08%)	6(2.47%)	4(4.88%)	1.19	0.275

#### 3.2.1 Preoperative factors

Fourteen patients (17.1%) in the prolonged group experienced preoperative bowel obstruction, significantly higher than the 21 patients (8.6%) in the normal group.

The incidence of preoperative perforation was also significantly higher in the prolonged group (10 patients, 4.1% in the normal group vs. a higher proportion in the prolonged group, with a statistically significant difference).

#### 3.2.2 Surgical-related factors

There was no significant difference in the distribution of surgical sites (left vs. right colon) between the two groups (left colon: 81 patients, 33.3% in the normal group vs. 27 patients, 33.0% in the prolonged group; right colon: 162 patients, 66.7% in the normal group vs. 55 patients, 67.0% in the prolonged group).

The proportion of Class IV incisions was significantly higher in the prolonged group (8 patients, 9.8% vs. 6 patients, 2.5% in the normal group).

The proportion of open surgery was significantly higher than laparoscopic surgery in the prolonged group (41 patients, 50.0% vs. 56 patients, 23% in the normal group).

Intraoperative blood loss was significantly higher in the prolonged group when compared with the normal group.

The median ventilator use time was 20.5 hours in the prolonged group, significantly longer than the 15 hours in the normal group.

#### 3.2.3 Postoperative complications

The incidence of perioperative sepsis was significantly higher in the prolonged group (21 patients, 25.6% vs. 4 patients, 1.6% in the normal group).

The incidence of perioperative acute kidney injury (AKI) was significantly higher in the prolonged group (12 patients, 14.6% vs. 3 patients, 1.2% in the normal group).

The incidence of lower extremity venous thrombosis was significantly higher in the prolonged group (16 patients, 19.5% vs. 24 patients, 9.9% in the normal group) (p< 0.05).

There were no statistically significant differences between the two groups in the incidence of postoperative wound infection (5 patients, 6.1% in the prolonged group vs. 13 patients, 5.3% in the normal group), postoperative wound dehiscence (3 patients, 3.7% in the prolonged group vs. 5 patients, 2.0% in the normal group), or postoperative pulmonary infection (4 patients, 4.9% in the prolonged group vs. 6 patients, 2.5% in the normal group). Similarly, there was no significant difference in the incidence of postoperative intestinal fistula (2 patients, 2.4% in the prolonged group vs. 4 patients, 1.7% in the normal group) (p > 0.05).

### 3.3 Comparison of mean laboratory results within the first 24 hours of ICU admission

The prolonged group had significantly lower platelet counts (PLT) and albumin levels (ALB) and significantly higher blood urea nitrogen (BUN) levels compared to the normal ICU stay group. No significant differences were observed in other indicators (such as WBC, Hb, ALT, total bilirubin, Scr, Na) between the two groups (**Table 3**). These findings suggest potential differences in nutritional status between the two groups.

**Table 3:** Comparison of average tests on the first day of ICU admission.

Variable	ALL (N = 325)	Normal group (N = 243)	Delayed group (N = 82)	X^2^/t	p
WBC	9.68(7.64,12.36)	9.79(7.64,12.23)	9.51(7.61,13.13)	0.005	0.94
PLT	205(154.66,256)	209.5(160,253)	176.9(131.2, 263.5)	5.17	0.023
Hb	104.5(93.5,116)	103.5(93.5,115.5)	106.5(92.5,117.5)	0.44	0.51
ALB	29.75(27.5,32.5)	30.05(28,32.7)	28.43(25.15,32)	10.39	0.001
ALT	20.75(15,28.5)	20.5(14.5,28.5)	22(15.5,29.5)	0.71	0.398
Bilirubin	17.15(12.1,24.05)	16.93(12.1,22.75)	18.6(12.1,25.55)	0.89	0.34
Scr	63(50.5,78)	63(51.5,77.5)	61.25(47,83)	0.06	0.81
Bun	4.58(3.48,6.48)	4.4(3.37, 6.5)	5.5(4,7.6)	9.67	0.002
Na	139.7(137.35,141.9)	139.7(137.5,141.85)	139.3(136.9,142.9)	0.007	0.935

### 3.4 Risk factor analysis for prolonged ICU stay after colorectal cancer surgery

After adjusting for preoperative bowel obstruction, preoperative perforation, Class IV incisions, open surgical approach, intraoperative blood loss, ventilator use time, perioperative sepsis, perioperative AKI, lower extremity venous thrombosis, and the average values of platelet count, albumin, and blood urea nitrogen within the first 24 hours of ICU admission, multivariate logistic regression analysis revealed that an open surgical approach, perioperative sepsis, prolonged ventilator use time, the occurrence of lower extremity venous thrombosis, and the average platelet count within the first 24 hours of ICU admission were independent risk factors for prolonged ICU stay (**Table 4**). The variance inflation factors (VIFs) for all variables were less than 10, indicating no significant collinearity.

**Table 4:** Multivariate regression results.

Variable	Odds ratio	Std. err.	z	P>z	[95% conf. interval]
PLT	0.99	0.002	-2.04	0.041	0.9917, 0.9998
Laparotomy	2.19	0.782	2.21	0.027	1.0913, 4.4136
Mechvent	1.027	0.0072	3.88	0.0001	1.0135, 1.041
Sepsis	1.4	1.2	2.96	0.003	1.452, 8.23
DVT	2.31	0.949	2.04	0.042	1.0322, 5.169
_cons	2.668794	0.33	-1.06	0.290	0.23, 3.08

### 3.5 ROC curve analysis

**Figure 1:**
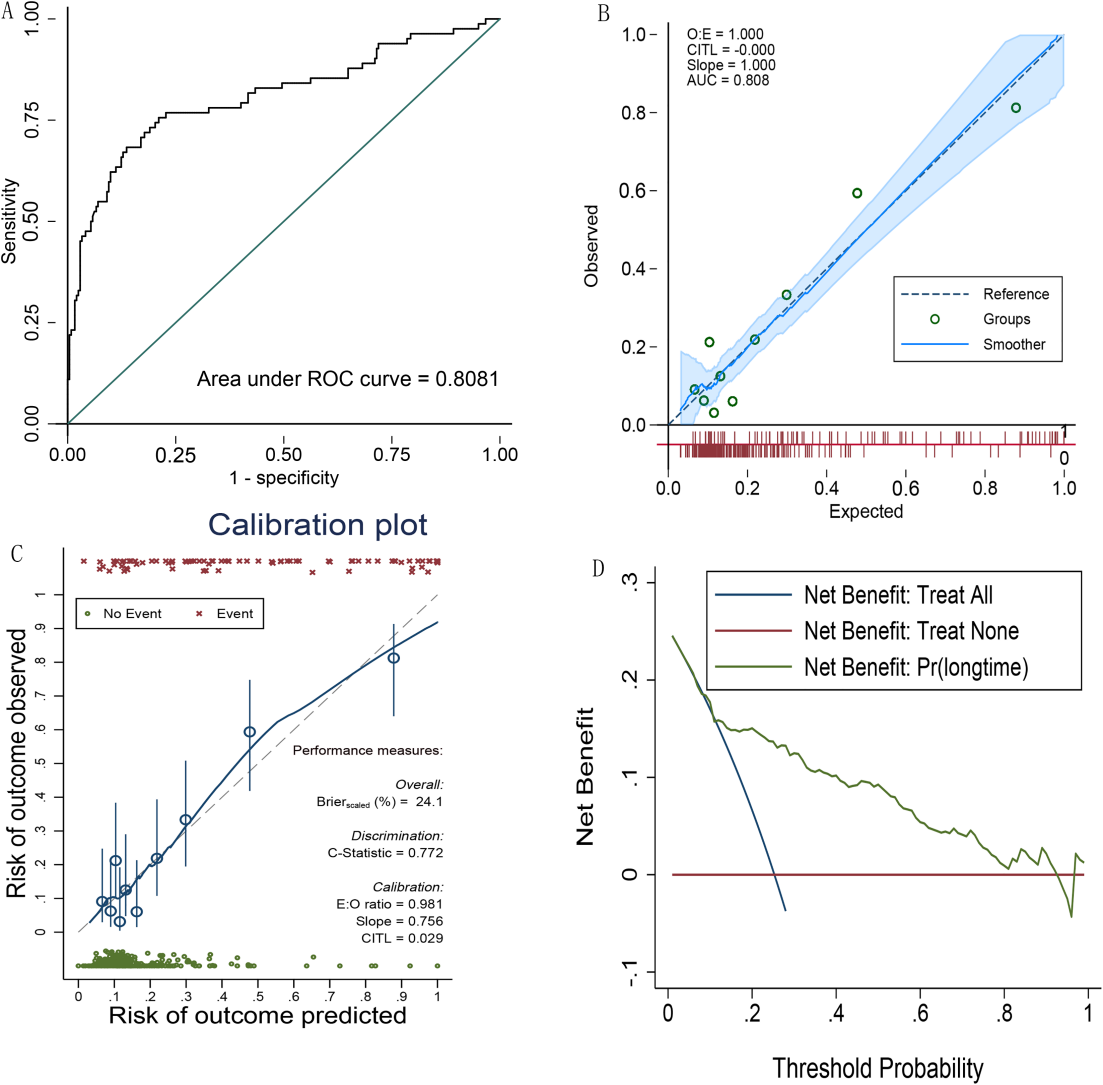
**(A)** The area under the ROC curve (AUC) of model; **(B)** the calibration curve of model; **(C)** bootstrap analysis results; **(D)** decision curve analysis of model.

**Figure 1** shows that the area under the ROC curve (AUC) was 0.8081 (95% CI: 0.745-0.87) (**Figure 1A**). The calibration curve demonstrated good agreement between the predicted and observed outcomes of prolonged ICU stay (O:E = 1.000, CITL = -0.000, Slope = 1.000), with strong discriminatory ability (AUC = 0.808) (**Figure 1B**). Bootstrap analysis revealed a Brier score of 24.1%, a C-statistic of 0.772, an E:O ratio of 0.981, a slope of 0.756, and a CITL of 0.029 (**Figure 1C**). Decision curve analysis indicated that the model for predicting prolonged ICU stay after colorectal cancer surgery provided a high net benefit at lower threshold probabilities (**Figure 1D**).

### 3.6 Subgroup analysis

**Figure 2:**
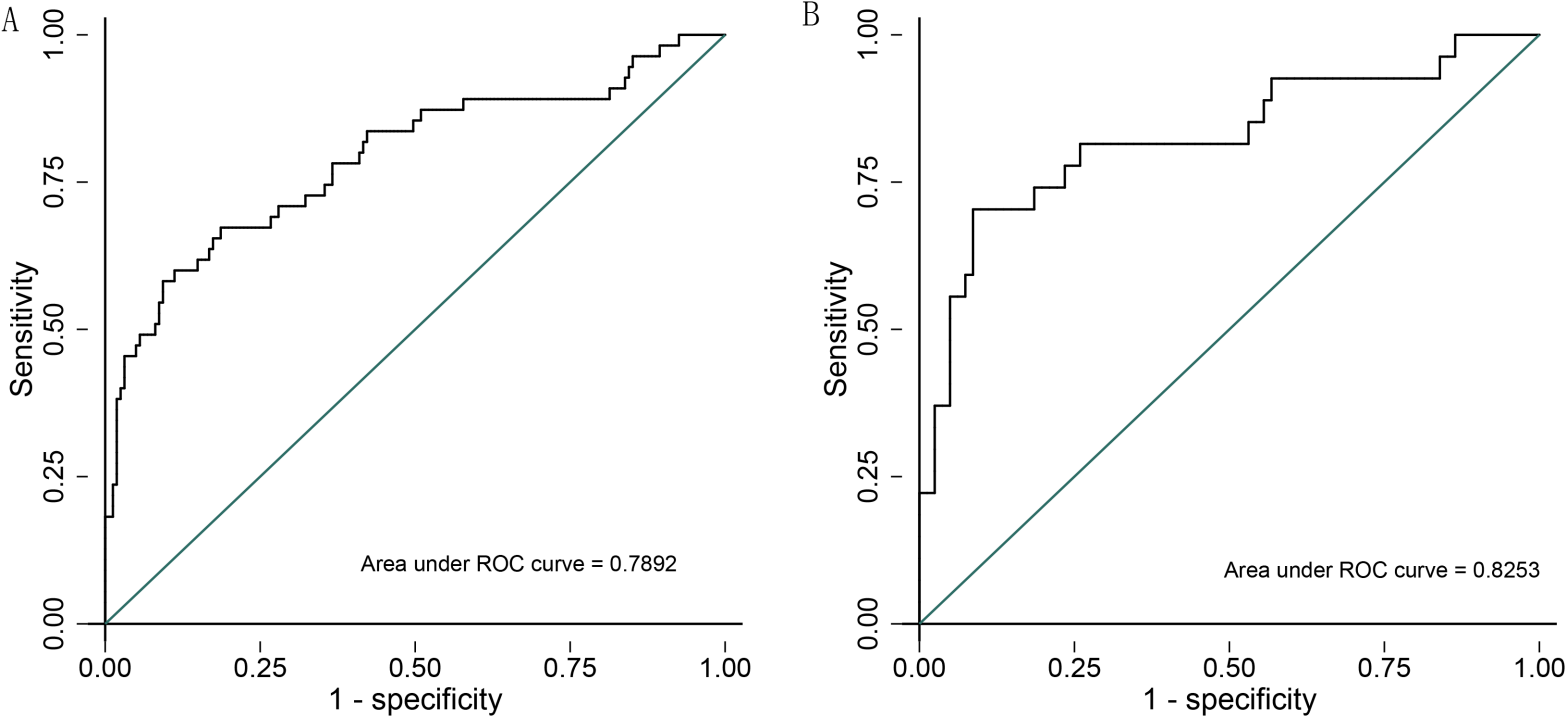
Subgroup analysis based on the surgical site. **(A)** the AUC for left colon subgroup; **(B)** the AUC for right colon subgroup.

Subgroup analysis based on the surgical site (left vs. right colon) showed that the AUC for the left colon subgroup was 0.7892 (95% CI: 0.71-0.87) (**Figure 2A**), and for the right colon subgroup, it was 0.8253 (95% CI: 0.72-0.93) (**Figure 2B**).

## 4 Discussion

This study identified independent risk factors for prolonged ICU stay following colorectal cancer surgery, including an open surgical approach, perioperative sepsis, prolonged ventilator use time, the occurrence of lower extremity venous thrombosis, and lower average platelet counts within the first 24 hours of ICU admission.

The decision to admit oncology patients to the ICU is typically made based on the cancer diagnosis and the presence of complications. Recent research has shown that tumor staging is not associated with in-hospital mortality during ICU treatment (9). Consistent with this finding, our study found no correlation between prolonged ICU stay and tumor staging. The significantly higher incidence of postoperative complications in the prolonged ICU stay group compared to the normal ICU stay group may be attributed to multiple interacting factors. These include preoperative pathological status, surgical complexity, intraoperative blood loss, ventilator use time, and the patients’ overall health status. Collectively, these factors contribute to an increased risk of postoperative complications.

The higher proportion of preoperative bowel obstruction and perforation in the prolonged ICU stay group may place patients in a compromised physiological state prior to surgery, thereby increasing the difficulty and risk of the procedure, and consequently, the incidence of postoperative complications. The elevated rate of Class IV incisions in the prolonged ICU stay group, which typically indicates greater surgical complexity and trauma, can lead to more challenging postoperative recovery and a higher likelihood of complications. Additionally, the higher proportion of open surgical procedures in the prolonged ICU stay group, which are associated with greater trauma, longer recovery times, and higher complication rates compared to laparoscopic surgery, further contributes to this trend. The increased incidence of intraoperative blood loss ≥50ml in the prolonged ICU stay group may result in postoperative anemia, hypotension, and other conditions that impede recovery and increase the risk of complications. Prolonged ventilator use may also elevate the risk of respiratory complications, such as pulmonary infections.

The significantly higher rates of perioperative sepsis and acute kidney injury (AKI) in the prolonged ICU stay group may be linked to preoperative pathological status, surgical complexity, and intraoperative blood loss. These complications exacerbate the patient’s condition and prolong recovery time. Open surgery, compared to laparoscopic surgery, is associated with greater trauma and postoperative complications, which can lead to prolonged hospital stays (10). Meta-analyses have indicated that patients undergoing open surgery have significantly longer postoperative hospital stays (OR = 3.35) (10) and that open surgery extends postoperative hospital stays by 2–3 days and ICU stays by 1.5-fold compared to laparoscopic surgery (11). Previous studies have also highlighted the advantages of laparoscopic surgery in reducing postoperative complications and shortening hospital stays (12), with less need for postoperative ICU support. Despite these benefits, surgeons may opt for open surgery in cases of anticipated complexity or unexpected intraoperative challenges. Prior research has confirmed that the surgical approach (open vs. laparoscopic) is a risk factor for postoperative ICU admission in colorectal cancer patients (5). Our study further demonstrates that the surgical approach is also a risk factor for prolonged ICU stay in these patients, emphasizing the importance of selecting an appropriate surgical method to reduce ICU stay duration.

Despite advancements in oncological care, tumor-related complications such as bowel obstruction, intestinal perforation, sepsis, and acute kidney injury continue to pose challenges for oncologists and critical care specialists (13). Notably, cancer patients with septic shock do not exhibit increased in-hospital or ICU mortality rates compared to non-cancer patients (14). However, perioperative sepsis remains a significant factor contributing to prolonged ICU stay following colorectal cancer surgery. Postoperative sepsis in colorectal cancer often arises from intra-abdominal infections, anastomotic leaks, or bacterial translocation, leading to multi-organ dysfunction. Sepsis, identified as the strongest independent risk factor for prolonged ICU stay (OR = 4.2), triggers systemic inflammatory response syndrome (SIRS), multi-organ dysfunction, and immunosuppression (15). It exacerbates inflammatory responses and may cause multi-organ failure, thereby extending ICU stays. This aligns with existing literature on the detrimental effects of sepsis on postoperative recovery. Preoperative perforation and obstruction in colorectal cancer patients can precipitate intra-abdominal infections, with sepsis potentially masking the direct effects of perforation in multivariate models. Similarly, Class IV (contaminated) incisions may indirectly influence ICU stay duration by increasing infection risks, although their effects may be overshadowed by the more direct pathophysiological processes of sepsis.

Prolonged postoperative ventilator use is an independent predictor of prolonged ICU stay (OR = 2.8) (5). Extended ventilator use is often associated with impaired pulmonary function or postoperative complications. Prolonged mechanical ventilation increases the risk of ventilator-associated pneumonia and other complications, thereby prolonging ICU stays. Postoperative respiratory insufficiency in colorectal cancer patients may be linked to prolonged anesthesia times, postoperative pain restricting respiratory movements, or pulmonary infections. Optimizing postoperative respiratory management and reducing ventilator use time are critical measures to shorten ICU stays.

Postoperative venous thromboembolism (VTE), a major and potentially fatal complication of colorectal cancer surgery (16), is associated with tumor hypercoagulability, surgical trauma, and postoperative immobilization. Cancer patients admitted to the ICU postoperatively are at higher risk of lower extremity venous thrombosis compared to non-cancer patients, which may exacerbate systemic inflammatory responses and prolong ICU stays (17). Prophylactic anticoagulation therapy and early mobilization are key strategies to reduce the incidence of lower extremity venous thrombosis. Our study identified lower extremity venous thrombosis as a risk factor for prolonged ICU stay in postoperative colorectal cancer patients.

Lower platelet counts within the first 24 hours of ICU admission (<100×10^9/L) are independently associated with prolonged ICU stays (OR = 1.9) (18). Thrombocytopenia may result from sepsis-associated disseminated intravascular coagulation (DIC), bone marrow suppression, or drug toxicity. Lower average platelet counts within the first 24 hours of ICU admission may reflect postoperative coagulation abnormalities or occult bleeding. Thrombocytopenia is closely linked to sepsis, DIC, and multi-organ failure. Additionally, platelets play a role in inflammatory regulation, and abnormal platelet levels may exacerbate systemic inflammatory responses, impairing organ repair. Our study demonstrated a correlation between lower average platelet counts within the first 24 hours of ICU admission and prolonged ICU stays in postoperative colorectal cancer patients.

Severe complications are associated with prolonged ICU stay. Patients with colorectal cancer often face weakness due to the disease itself and treatment side effects (such as surgery, chemotherapy, malnutrition). Prolonged mechanical ventilation and sedative exposure, leading to ICU-acquired weakness and delirium. The hospitalization experience in the ICU may further exacerbate weakness (19). Preoperative nutritional status, laboratory results such as white blood cell count, liver and kidney function, and the duration of gastrointestinal perforation may also affect the length of ICU stay. Immunoparalysis and persistent systemic inflammation after AKI, predisposing to secondary infections. Non-clinical reasons remain a significant contributor to delayed discharge in a proportion of patients with no postoperative complications. Implementation of a nurse-led “DVT-prevention checklist” may reduce DVT incidence. Awakening-and-Breathing Coordination (ABC) protocol may decrease median ventilation time. Enhanced recovery after surgery (ERAS) protocols are multimodal perioperative care pathways. Implementation of the ERAS protocol for colorectal cancer led to a substantial improvement in compliance and a reduction in LOS, without meaningful effects on complications (20).

### 4.1 Limitations

This study is a single-center retrospective analysis, which may be subject to selection bias. Future multi-center prospective studies are warranted to validate our findings. Additionally, exploring the value of promoting minimally invasive surgery, implementing early warning systems for sepsis, and developing individualized anticoagulation regimens in shortening postoperative ICU stays for colorectal cancer patients is essential.

## 5 Conclusion

In summary, laparotomy, perioperative sepsis, prolonged ventilator use time, occurrence of lower extremity venous thrombosis, and lower average platelet counts within the first 24 hours of ICU admission are risk factors for prolonged ICU stay following colorectal cancer surgery. Therefore, in clinical practice, it is essential to identify, prevent, and control these risk factors early in such patients.

## Data Availability

The raw data supporting the conclusions of this article will be made available by the authors, without undue reservation.
